# Prey Preference of the Freshwater Jellyfish *Craspedacusta sowerbii*: Integrated Behavioral Observations and Transcriptomic Responses to Phytoplankton and Zooplankton

**DOI:** 10.3390/biology15212002

**Published:** 2026-11-04

**Authors:** Hailong Yan, Yu Wang, Lin Li, Yufan He, Jinxiang Han, Kefei Zhu, Yingchun Gong, Nicola Fohrer, Jianguang Qin, Yuying Li

**Affiliations:** 1 International Joint Laboratory of Watershed Ecological Security for Water Source Region of Middle Route Project of South-North Water Diversion in Henan Province, College of Water Resource and Modern Agriculture, Nanyang Normal University, Nanyang 473061, China; lilin130719@163.com (L.L.); 15637509979@163.com (Y.H.); hanjinxiang2026@163.com (J.H.); 13673396417@163.com (K.Z.); nfohrer@hydrology.uni-kiel.de (N.F.); lyying200508@163.com (Y.L.); 2 Henan Academy of Fishery Sciences, Henan Academy of Agricultural Sciences, Zhengzhou 450044, China; wangyu@hnagri.org.cn; 3 Institute of Hydrobiology, Chinese Academy of Sciences, Wuhan 430072, China; 4 Department of Hydrology and Water Resources Management, Kiel University, 240980 Kiel, Germany; 5 College of Science and Engineering, Flinders University, Adelaide, SA 5001, Australia; jian.qin@flinders.edu.au

**Keywords:** *Craspedacusta sowerbii*, invasive alien species, phytoplankton, prey preference, RNA-seq, zooplankton

## Abstract

Freshwater ecosystems are highly vulnerable to biological invasions. The globally distributed invasive hydrozoan *Craspedacusta sowerbii* imposes strong top-down pressure on plankton communities owing to its distinct feeding selectivity. While prior studies have confirmed its preferential predation on zooplankton over phytoplankton, the integrated mechanisms underlying this prey discrimination remain poorly understood. Here, we integrated behavioral observations and comparative transcriptomics to investigate the prey preference strategy of adult *C. sowerbii* medusae. Behavioral assays demonstrated active predation exclusively on zooplankton, with no targeted hunting toward phytoplankton. Microscopic observations revealed efficient degradation of ingested zooplankton within 6 h, whereas phytoplankton cells were excreted with no apparent digestion. Transcriptomic analyses indicated that zooplankton feeding induces profound transcriptional reprogramming in *C. sowerbii*. Genes related to catalytic and hydrolase activities were significantly enriched, accompanied by substantial up-regulation of ribosome biogenesis and mitochondrial oxidative phosphorylation. In contrast, phytoplankton exposure triggered negligible metabolic perturbation. The coordinated activation of hydrolase, protein synthesis, energy metabolism, cytoskeletal movement, and mucus production forms the potential physiological basis for the feeding preference of *C. sowerbii*. Field observations further suggest that algicidal bacteria in the gastric cavity of medusae may assist auxiliary phytoplankton utilization. This study elucidates the regulatory mechanisms governing the trophic selectivity of *C. sowerbii*, fills key knowledge gaps in its feeding ecology, and offers new insights into the environmental adaptability and survival strategies of this species.

## Simple Summary

Freshwater environments are easily damaged by non-native invasive species. The globally spread freshwater jellyfish *Craspedacusta sowerbii* disturbs plankton communities through its distinct food-selection habits. Prior studies show it prefers zooplankton over phytoplankton, yet little is known about what drives this preference. Here, we combined feeding behavior observations and RNA-seq comparisons on adult jellyfish to investigate their food-choosing patterns. Behavioral assays demonstrated that the jellyfish actively hunt zooplankton but do not target phytoplankton. Microscopic observations revealed ingested zooplankton prey breaks down fully within 6 h, while phytoplankton prey remains undigested. Feeding on zooplankton triggers widespread changes in the transcriptome, activating genes involved in food breakdown, protein synthesis and energy generation. Exposure to phytoplankton causes almost no such transcriptional changes. The integrated effects of specific activation of digestive enzymes, protein synthesis, energy supply, body movement and mucus secretion likely support the different feeding behaviors of the jellyfish. Field observations further suggest that stomach-associated algicidal bacteria may help the jellyfish make limited use of phytoplankton. This study reveals how the jellyfish regulates its food preference and provides new insights into its environmental adaptation and survival strategy.

## 1. Introduction

Invasive alien species (IAS) are non-native organisms that colonize new ecosystems via natural or anthropogenic pathways, establish self-sustaining populations, and proliferate rapidly under favorable conditions [1,2,3]. While a subset of alien species can deliver measurable socioeconomic benefits to local communities [4,5], most IAS exert strong suppressive pressure on native species through predation, competitive exclusion and habitat modification, driving native biodiversity loss and cascading ecosystem degradation [6,7,8]. These disturbances pose severe, multi-faceted threats to regional biodiversity integrity [3,9], fisheries and aquaculture productivity [7,8], public health security [4,10], and long-term economic sustainability [2,5]. Globalization and climate change have substantially accelerated IAS dispersal, leading to a sharp rise in invasive events worldwide [9,11]. Over 37,000 alien species have been documented, and 3500 of them are recognized as harmful invasive species [12]. These invaders cause annual economic losses of over $423 billion, establishing IAS as one of the five primary direct drivers of global ecosystem change over the past 50 years [2,12]. Driven by pervasive hydrological connectivity and the inherent ecological vulnerability, aquatic ecosystems exhibit disproportionately greater susceptibility to biological invasions and are widely acknowledged as invasion hotspots [8,13]. Aquatic and semi-aquatic taxa account for approximately one-quarter of the 100 World’s Worst Invasive Alien Species, highlighting the urgent need to elucidate the fundamentals of aquatic biological invasions for safeguarding water security [4,6,14].

The freshwater jellyfish *Craspedacusta*, a cnidarian genus belonging to the class Hydrozoa, order Limnomedusae, and family Olindiidae, is native to the Yangtze River Basin, China [15,16]. A total of 11 taxonomic units (including varieties) have been formally documented, with valid species comprising *C. sowerbii*, *C. sinensis*, and *C. iseanum* [15,17,18]. Long categorized in China as a rare aquatic group [16], this genus has recently been identified as a globally invasive taxon owing to its extensive cross-regional dispersal and adaptive colonization capacity [19,20,21]. The species *C. sowerbii*, first recorded by W. Sowerby in 1880 from the Royal Botanic Gardens, Regent’s Park, London [22], has expanded rapidly into higher-latitude regions under climate warming. It is now confirmed to occur on all continents except Antarctica and has been documented as an invasive species across Europe, North America, and parts of Asia, where it colonizes diverse freshwater habitats including lakes, reservoirs, ponds, slow-flowing rivers, and artificial impoundments [20,23,24]. As apex invertebrate predators, blooming jellyfish often consume a huge amount of plankton and further cause deleterious consequences for local fisheries [25,26]. *Craspedacusta sowerbii* has the potential to reshape food web structure via intensive predation on copepods, rotifers and other zooplankton and imposes strong competitive pressure on larger zooplankton and juvenile fish sharing identical prey resources [27,28]. Full-grown *Craspedacusta* medusae can consume up to 190 zooplankton within 4–5 h [29]. Such high predation efficiency significantly modulates the structure of local zooplankton communities [19,28]. The resultant top-down trophic disturbances alter energy flow, thereby generating substantial adverse impacts on ecosystem stability [27,30]. Accordingly, elucidating the invasion mechanisms and survival strategies of *Craspedacusta* is critical for clarifying its ecological impacts and establishing targeted management tactics for aquatic ecological sustainability.

Multiple environmental factors regulate the growth of *Craspedacusta* either independently or synergistically [31,32,33], among which nutrient availability serves as a core determinant of population survival [32,34]. Although both phytoplankton and zooplankton can serve as potential food resources for *Craspedacusta* [16], integrated evidence from field monitoring and microscopic gastric content analysis confirms that this species exhibits distinct feeding selectivity toward zooplankton [29,35]. Specifically, cladocerans and copepods constitute its preferred prey [36], and the medusa preferentially preys on larger zooplankton (0.4–1.4 mm) rather than small ones [24,29]. Furthermore, amplicon sequencing combined with in vivo observations reveals that in the reservoir environment, zooplankton occupy a significantly higher relative abundance in the gastric cavity of *Craspedacusta* than phytoplankton [16]. Consistent with this feeding pattern, trophic ecological studies on *Hydra vulgaris*, a closely related hydrozoan species, have demonstrated that despite the numerical dominance of phytoplankton in gut contents, their volumetric contribution and nutritional value are negligible [37]. The prevailing hypothesis attributes this selective feeding to the specialized inter-nematocyst spacing on *Craspedacusta* tentacles, a morphological trait optimized for capturing large prey [29], whereas phytoplankton are mostly ingested passively with water flow [37]. However, this hypothesis fails to explain why *Craspedacusta* can ingest but cannot digest phytoplankton. Although *Craspedacusta* has been studied for almost 150 years since its initial documentation, most prior work focuses merely on morphological taxonomy and behavioral traits; its physiology and fundamental metabolic regulatory pathways remain largely unknown [18,38].

To unravel the physiological basis underlying the differential feeding responses of *Craspedacusta* to zooplankton and phytoplankton, the present study adopted adult *C. sowerbii* medusae as experimental materials. We established an indoor artificial culture system for *C. sowerbii* and performed controlled feeding experiments with zooplankton and phytoplankton as separate diets. By integrating behavioral observations and comparative transcriptomic profiling, we explored the prey preference of *C. sowerbii*. Our findings reveal that *C. sowerbii* actively captures and digests zooplankton while showing no targeted predation or digestion toward phytoplankton. Transcriptomic analysis indicated that zooplankton feeding remodeled abundant genes responsible for catalytic and hydrolase activities and activated conserved functional pathways, including ribosome biogenesis and mitochondrial energy metabolism, while phytoplankton exposure induced only trivial transcriptional alterations. This study systematically characterizes the physiological regulatory divergence underlying prey processing in *C. sowerbii* across distinct dietary conditions, fills a long-standing knowledge gap in the selective feeding ecology of *Craspedacusta*, and provides novel insights into the invasion success and trophic adaptation strategies of this globally distributed invasive freshwater jellyfish.

## 2. Materials and Methods

### 2.1. Sampling and Indoor Cultivation of Craspedacusta sowerbii

Samples were collected from a countryside pond (115.0708° E, 30.0583° N, Huangshi, China) where stable natural populations of *Craspedacusta* have persisted continuously for eight years. Species identity was molecularly confirmed as *C. sowerbii* via amplicon sequencing in a previous study [16]. Live medusae were gently collected using 10 L plastic buckets and temporarily maintained in in situ pond water before laboratory transportation. Upon arrival, all individuals were transferred to pre-treated tap water that had been fully dechlorinated through 48 h of static aeration. After 24 h of laboratory acclimation, specimens were transferred into fresh dechlorinated tap water to eliminate residual pond plankton. Phytoplankton and zooplankton were prepared as live prey items for *C. sowerbii*. Given that it feeds preferentially on zooplankton over phytoplankton [18,35], and to improve the algal ingestion rate, a mixture of *Haematococcus*, *Chlorella*, and *Scenedesmus* was prepared as phytoplankton prey. *Artemia* nauplii, which are easy to culture in the laboratory and can serve as suitable prey for jellyfish [39], were used as the zooplankton prey. Both algae strains and *Artemia* nauplii were maintained in our laboratory; all prey suspensions were freshly prepared and were homogenized in dechlorinated tap water prior to feeding. Indoor cultivation was maintained at a constant temperature of 25 °C under a 12 h:12 h light–dark photoperiod, with a photon flux density of 30 μmol/(m^2^·s). For biosecurity and ethical compliance, all post-experimental specimens of *C. sowerbii* were reared indoors with regular feeding until natural death.

### 2.2. Morphological Observation of the Feeding Behavior of C. sowerbii

**Figure 1:**
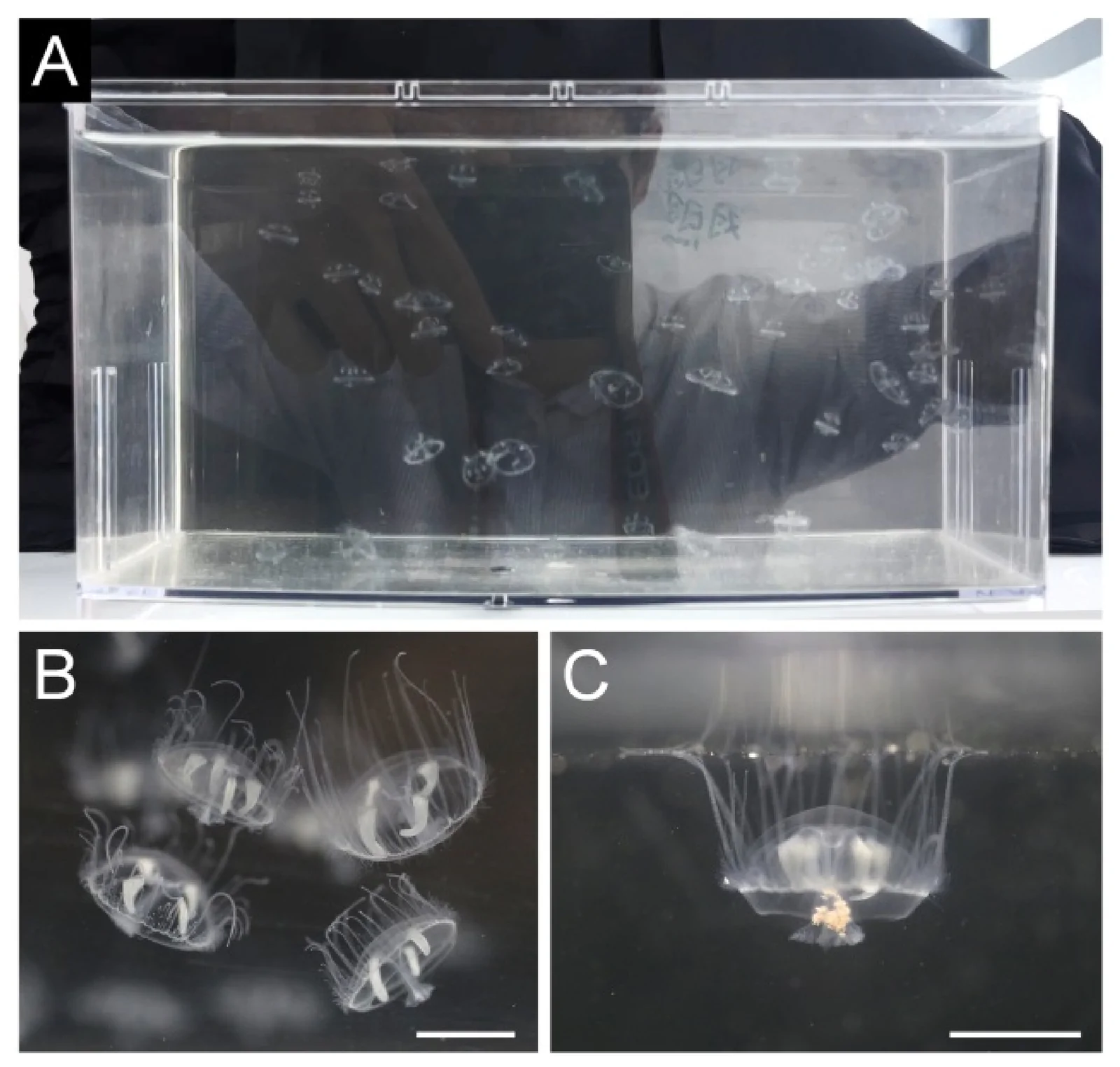
The culture system and morphological visualization of *Craspedacusta sowerbii*. (**A**) Indoor culture system using a 3-L glass aquarium. (**B**) Morphological characteristics of *C. sowerbii*. (**C**) In situ feeding behavior captured via macro photography. Bars = 1 cm.

All behavioral experiments were performed at 25 °C under consistent laboratory conditions. A total of about 500 *C. sowerbii* medusae were randomly assigned to nine groups, with over 50 individuals per group. Each group was cultured in an independent 3 L glass aquarium (25 cm × 10 cm × 12 cm, Figure 1A). These groups were randomly divided into three treatments, including three blank control groups with no prey supplementation (CK), three phytoplankton-feeding groups (PHY), and three zooplankton-feeding groups (ART). Based on our rearing experience, the PHY groups were supplied with 5 mL of mixed algal suspension at final densities of about 5 × 10^5^ cells/mL *Haematococcus*, 1 × 10^7^ cells/mL *Chlorella*, and 1 × 10^7^ cells/mL *Scenedesmus*. The ART groups were fed 5 mL of *Artemia* nauplii suspension at a density of 10^3^ ind./mL. During feeding, prey was drawn into a pipette and slowly released 1–2 cm above the aquarium, allowing it to disperse gradually upon entering the water. To minimize the observational errors caused by continuous feeding, the culture water was fully replaced at 30 min post-feeding. Macroscopic feeding behaviors were recorded using a Canon 80D digital camera equipped with a 50 mm f/2.5 macro lens (Canon, Tokyo, Japan), which enabled high-resolution visualization of the translucent medusae (Figure 1B,C). For morphological observations, active individuals were randomly sampled at 10 min, 3 h, and 6 h post-feeding. Specimens were gently collected using a wide-bore pipette (10 mm tip diameter), placed on glass slides, and manually unfolded to expose the gastric cavity. Morphological and digestive characteristics were observed and photographed under an Olympus BX53 microscope coupled with a Canon 80D camera (Olympus, Tokyo, Japan). For each treatment and time point, no fewer than 25 individuals were observed to ensure biological representativeness. All photographic images were uniformly contrast-enhanced using Adobe Photoshop CC 64 Bit (Adobe Systems Incorporated, San Jose, CA, USA) for clearer morphological presentation.

### 2.3. Sample Preparation for RNA-Seq Analysis

Obvious intergroup differences in feeding performance and digestive morphology were consistently observed at 6 h post-feeding; therefore, this time point was selected for transcriptomic sampling. Fifteen individuals were randomly collected from each group at 6 h post-feeding to generate sufficient biological material for RNA sequencing. Sampled medusae were gently transferred into 15 mL cryotubes, and surface residual water was rapidly absorbed using sterile filter paper to avoid sample dilution. All samples were immediately frozen in liquid nitrogen and stored at −80 °C for subsequent RNA extraction. Total RNA was isolated using TRIzol reagent (Invitrogen, Carlsbad, CA, USA) according to the manufacturer’s standard protocol. RNA concentration and purity were determined using a NanoDrop 2000 spectrophotometer (Thermo Fisher Scientific, Waltham, MA, USA), and RNA integrity was verified using an Agilent 2100 Bioanalyzer with an RNA Nano 6000 Assay Kit (Agilent Technologies, Santa Clara, CA, USA). Qualified RNA samples with high purity and integrity were reverse-transcribed into double-stranded cDNA and sequenced on an Illumina NovaSeq X Plus platform (Illumina, San Diego, CA, USA) with a 2 × 150 bp paired-end strategy. Library construction and high-throughput sequencing were performed by Novogene Co., Ltd. (Beijing, China).

### 2.4. Bioinformatics Analysis Pipeline

Raw FASTQ sequencing reads were preprocessed using fastp (v0.23.4) to remove adapter sequences, poly-N reads, and low-quality reads, yielding high-quality clean reads for subsequent analysis. De novo transcriptome assembly was performed using Trinity (v2.15.1). Assembled transcripts were further clustered and refined using Corset (v1.09) to eliminate redundant sequences, and each final cluster was defined as a unigene. The completeness and quality of the assembled transcriptome were evaluated using BUSCO (v3.0.2) based on eukaryotic universal single-copy orthologs. Functional annotation of unigenes was performed by sequence alignment against seven public databases: NCBI Nr and Nt, Pfam, KOG, Swiss-Prot, Gene Ontology (GO), and Kyoto Encyclopedia of Genes and Genomes (KEGG). The Trinity-assembled transcriptome was used as the reference genome for read mapping. Clean reads were aligned to the reference using RSEM (v1.3.3) with the strict bowtie2 parameter “mismatch 0” to ensure high-precision mapping. Reads with mapping quality (MQ) < 10, unpaired reads, and multi-mapping reads were filtered out to guarantee mapping accuracy.

### 2.5. Differential Expression Analysis

Differential gene expression analysis between paired groups was performed using the DESeq2 R package (v1.42.0), which adopts negative binomial generalized linear models to identify differentially expressed genes (DEGs). The Benjamini–Hochberg algorithm was applied to correct *p*-values and control the false discovery rate (FDR). Genes with *p* ≤ 0.05 and |log2FC| ≥ 1 were defined as significantly differentially expressed. Principal component analysis (PCA) was performed to assess the similarity of gene expression profiles across samples. Venn diagrams were generated to visualize the overlaps of DEGs among different treatments, and volcano plots were constructed to display the significance and magnitude of gene expression changes between pairwise comparisons. GO functional enrichment analysis of DEGs was implemented using the GOseq R package to eliminate gene length bias. GO terms with adjusted *p* ≤ 0.05 were considered statistically enriched. KEGG is a core public database that integrates multi-omics molecular information to systematically annotate biological pathways and cellular functions. In this study, KOBAS (v3.0) was used to perform KEGG pathway enrichment analysis based on the hypergeometric test. Pathways with adjusted *p* (padj) ≤ 0.05 were identified as significantly enriched. Notably, all GO and KEGG functional terms were annotated based on cross-species conserved databases. These annotated pathway names do not indicate the presence of vertebrate-specific structures or physiological systems in *C. sowerbii*; instead, they reflect universally conserved molecular modules underlying core metabolic and cellular processes across eukaryotes.

## 3. Results

### 3.1. Different Feeding Behavior of Craspedacusta sowerbii Toward Phytoplankton and Zooplankton

**Figure 2:**
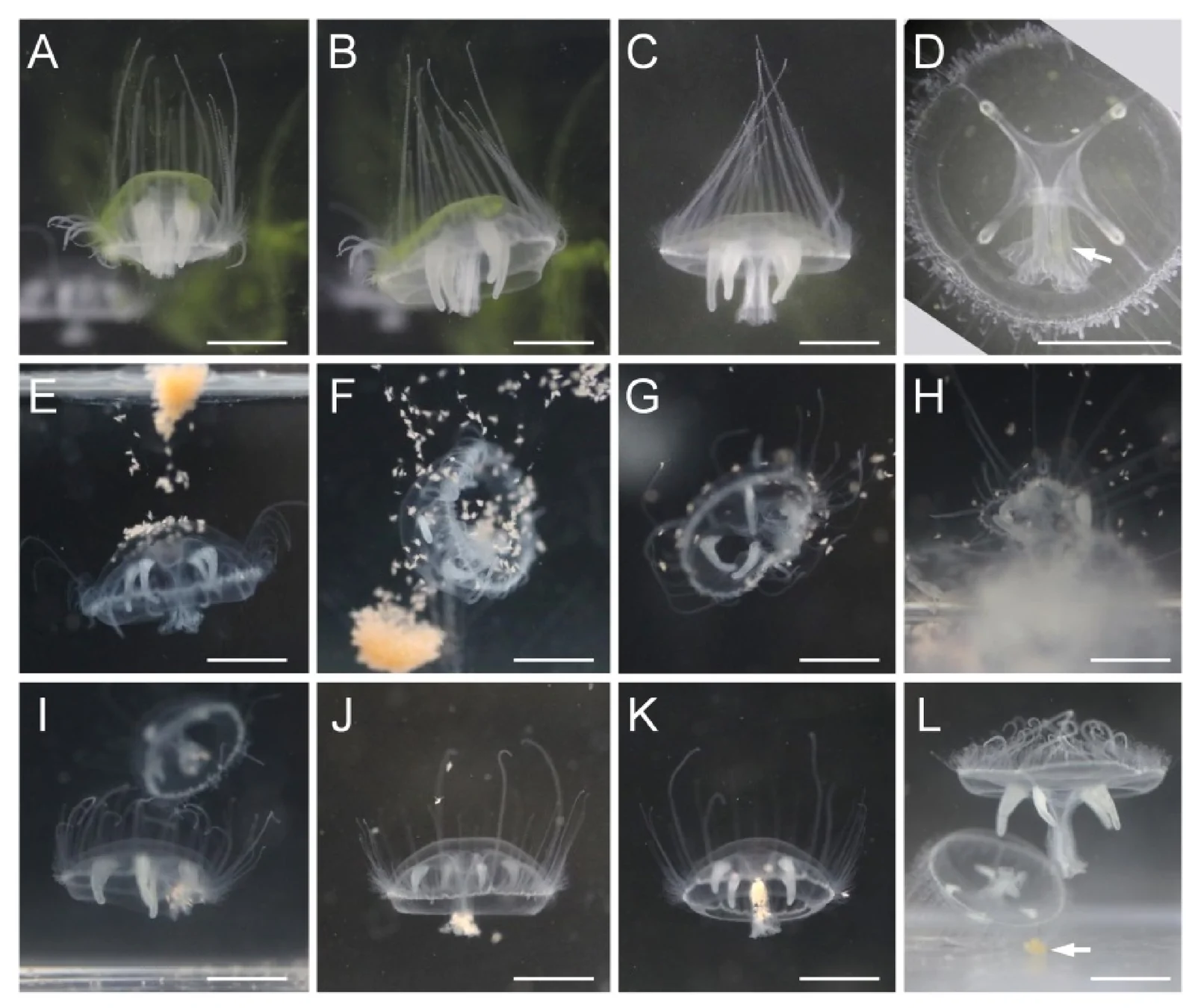
Feeding behavioral responses of *Craspedacusta sowerbii* to phytoplankton and zooplankton. (**A**–**C**) *C. sowerbii* shows no active predatory behavior during phytoplankton exposure. (**D**) Passively ingested phytoplankton cells within the gastric cavity (arrow). (**E**–**G**) Active predatory behaviors of *C. sowerbii* toward zooplankton. (**H**) Post-predation sinking behavior after prey enclosure. (**I**–**K**) *C. sowerbii* swam upward after fully ingesting zooplankton into the gastric cavity and continued to capture additional prey. (**L**) Expelled zooplankton residues (arrow). Bars = 0.5 cm.

Following the release and dispersion of phytoplankton in the culture water, *C. sowerbii* displayed no obvious behavioral preference or predatory interest toward the algal prey throughout the feeding experiment, even when algal cells settled on the medusa bell or accumulated adjacent to the tentacles (Figure 2A,B). This state remained consistent even after algal cells were evenly dispersed throughout the water column (Figure 2C). Notably, although no active predatory behavior was detected, residual green algal cells were observable within the gastric cavity of *C. sowerbii* (Figure 2D, arrow). This passive ingestion was most likely driven by the inhalant water currents produced by rhythmic bell pulsation, which entrapped suspended algal cells toward the oral arms, potentially assisted by surface mucus adhesion on the bell epidermis.

In sharp contrast, *C. sowerbii* exhibited intense and active predatory behavior toward zooplankton prey. Once zooplankton suspensions were released, medusa specimens rapidly initiated directional swimming toward prey aggregates (Figure 2E). Physical contact between prey and the medusa bell or tentacles triggered an immediate response, including body inversion and bell curling to enclose prey organisms (Figure 2F), followed by tight wrapping via oral arms (Figure 2G), and subsequently sinking slowly to the aquarium bottom (Figure 2H). After complete ingestion of zooplankton into the gastric cavity, *C. sowerbii* actively swam upward and resumed continuous prey capture (Figure 2I–K), repeating the cycle until the gastric cavity was fully occupied. Statistically, the mean number of zooplankton ingested in the gastric cavity was nearly 63 per medusa. At approximately 6 h post-feeding or later, internal zooplankton residues were excreted and deposited at the bottom of the aquarium (Figure 2L, arrow), leaving the gastric cavity empty.

### 3.2. Comparison of Digestive Responses of C. sowerbii Toward Phytoplankton and Zooplankton

**Figure 3:**
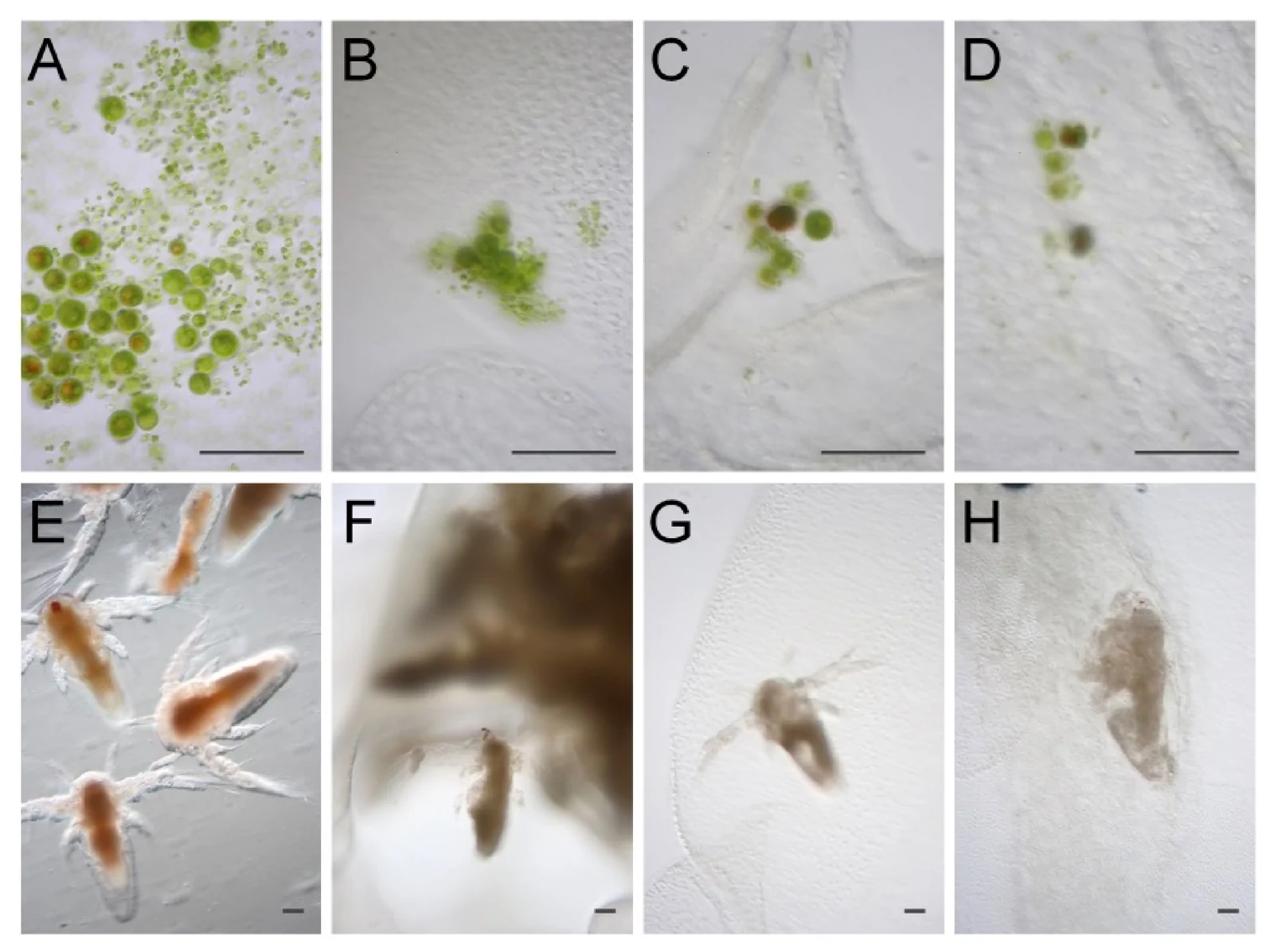
Dynamic digestive responses of *Craspedacusta sowerbii* toward phytoplankton and zooplankton. (**A**) Intact phytoplankton cells with complete cell walls and green pigmentation. (**B**) Aggregated phytoplankton cells inside the gastric cavity. (**C**,**D**) Morphological status of ingested phytoplankton at 3 and 6 h post-feeding, respectively. (**E**) Intact *Artemia* nauplii with distinct somatic structures. (**F**) Ingested *Artemia* nauplii clustered within the gastric cavity. (**G**,**H**) Degradation of *Artemia* nauplii at 3 and 6 h post-feeding, respectively. Note that phytoplankton cells remain structurally intact, while zooplankton individuals undergo substantial digestive decomposition. Bars = 100 μm.

Digestive capacity can more accurately reflect the actual prey utilization efficiency of *C. sowerbii* for different dietary resources. Therefore, medusae fed with phytoplankton or zooplankton were subjected to continuous microscopic observation, with a specific focus on the digestive status of prey within the gastric cavity. Phytoplankton cells presented regular spherical morphologies with abundant green pigments and intact cell wall boundaries (Figure 3A). After being passively ingested into the gastric cavity, phytoplankton cells aggregated into compact clusters and oscillated synchronously with the rhythmic pulsation of the gastric cavity (Figure 3B). At 3 h post-feeding, no obvious morphological degradation was observed, and the only detectable change was the gradual dispersion of aggregated cell clusters during gastric movement (Figure 3C). At 6 h post-feeding, the clustered phytoplankton cells were loosely distributed throughout the gastric cavity. Nevertheless, no digestive signs were identified, as the phytoplankton cells still retained full green pigmentation and completely intact cell walls (Figure 3D).

In contrast, *C. sowerbii* exhibited a high digestive efficiency toward zooplankton. Microscopic observation revealed that unfed *Artemia* nauplii possessed complete morphological characteristics, including distinct body outlines, low internal light transmittance, clearly segmented appendages, and intact somatic boundaries (Figure 3E). Once engulfed into the gastric cavity, *Artemia* nauplii immediately aggregated into clusters that moved passively with gastric pulsation (Figure 3F), consistent with the temporary aggregation pattern of phytoplankton cells. At 3 h post-feeding, the internal light transmittance of the nauplii increased significantly, accompanied by blurred somatic boundaries and progressive structural degradation, indicating the initiation of effective digestion by *C. sowerbii* (Figure 3G). By 6 h post-feeding, the majority of zooplankton bodies were thoroughly decomposed with indistinguishable morphological structures (Figure 3H), and the residual undigested fragments were subsequently excreted from the gastric cavity.

### 3.3. Transcriptomic Variations in C. sowerbii Fed with Phytoplankton or Zooplankton

**Figure 4:**
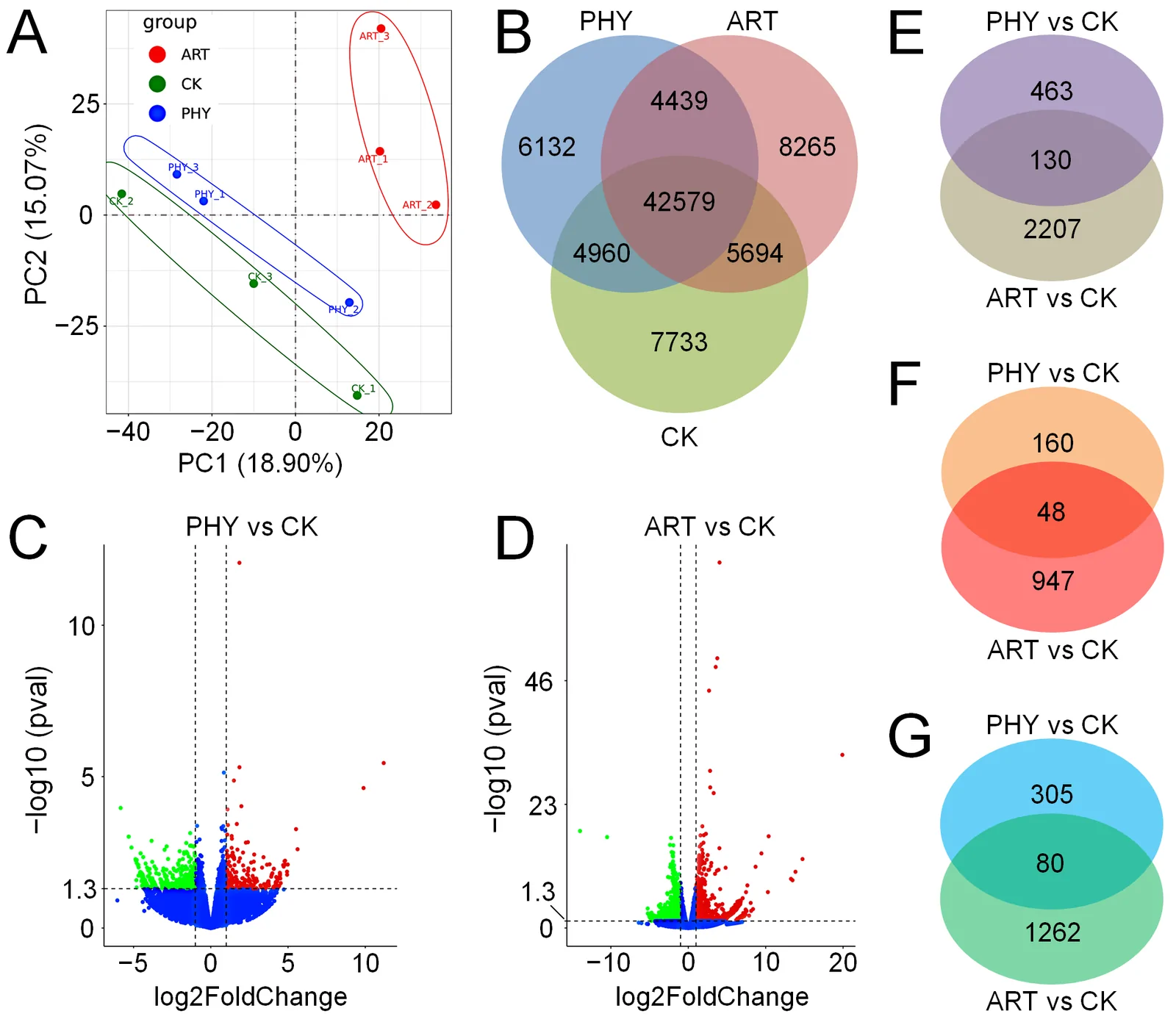
Overview of transcriptomic variations in *Craspedacusta sowerbii* under different prey treatments. (**A**) Principal component (PC) analysis showing transcriptional separation among control groups with no prey supplementation (CK), phytoplankton-feeding groups (PHY), and zooplankton-feeding groups (ART). (**B**) Venn diagram illustrating the overlap of all expressed genes across the three treatments. (**C**,**D**) Volcano plots displaying differentially expressed gene (DEG) distribution for PHY vs. CK and ART vs. CK comparisons, respectively. (**E**) Venn diagram showing unique and shared DEGs between comparative groups. (**F**,**G**) Statistical summary of up- and down-regulated DEGs in different comparisons, respectively.

Significant physiological and digestive differences were observed in *C. sowerbii* at 6 h post-feeding between phytoplankton-fed and zooplankton-fed medusae. Meanwhile, dietary residues within the gastric cavity were largely eliminated at this time point, which effectively minimized exogenous prey RNA contamination for subsequent transcriptomic profiling. Therefore, medusae from three distinct feeding treatments, including the CK, PHY, and ART groups, were sampled at 6 h post-feeding for comparative transcriptome analysis. Transcriptome assembly quality was validated via BUSCO in a previous study, confirming high assembly integrity and robustness for all downstream bioinformatic analyses [40]. PCA revealed distinct transcriptomic separation among three treatments. PC1 and PC2 explained 18.90% and 15.07% of the total transcriptional variance, respectively (Figure 4A). Specifically, the PHY groups exhibited a high degree of transcriptomic similarity and clustered closely with the CK groups, while the ART groups showed substantial transcriptional divergence from both the CK and PHY groups, indicating that different dietary prey types induced profound variations in the global gene expression profiles of *C. sowerbii*. RNA-seq annotation identified 60,966, 58,110, and 60,977 expressed genes in the CK, PHY, and ART groups, respectively. Among these, 47,539 genes were co-expressed in the PHY and CK groups, 48,273 genes were shared between the ART and CK groups, and 42,579 genes were expressed across all three treatments (Figure 4B, File S1).

Differential gene expression analysis demonstrated that the zooplankton prey triggered a much stronger transcriptional response than the phytoplankton prey. The ART vs. CK comparison yielded considerably more DEGs with higher fold-change magnitudes than the PHY vs. CK comparison (Figure 4C,D). In the PHY vs. CK comparison, a total of 593 DEGs were identified (Figure 4E, File S2), including 208 up-regulated and 385 down-regulated genes (Figure 4F,G). Whereas the ART vs. CK comparison produced 2337 DEGs (Figure 4E, File S2), consisting of 995 up-regulated and 1342 down-regulated genes (Figure 4F,G). Furthermore, 130 DEGs were commonly shared between the two comparative groups (Figure 4E, File S2), among which 48 genes were up-regulated and 80 genes were down-regulated (Figure 4F,G).

### 3.4. Metabolic Pathway Alterations in C. sowerbii Fed with Phytoplankton or Zooplankton

To elucidate the metabolic mechanisms underlying the divergent feeding and digestive responses of *C. sowerbii* to phytoplankton and zooplankton, GO functional classification and KEGG pathway enrichment analyses were performed on the DEGs derived from PHY vs. CK and ART vs. CK comparisons. Across all DEGs identified from the two comparative groups (2800 in total), functional GO annotation categorized 707 genes into Biological Process (BP), 804 into Molecular Function (MF), and 538 into Cellular Component (CC) (File S2). For the PHY vs. CK group (593 DEGs), 98 genes were annotated to BP, 91 to MF, and 66 to CC. Specifically, among the 208 up-regulated DEGs in the PHY vs. CK comparison, 45 belonged to BP, 36 to MF, and 31 to CC; and among the 385 down-regulated DEGs, 53 were classified as BP, 55 as MF, and 35 as CC. For the ART vs. CK comparison (2337 DEGs), 636 genes were assigned to BP, 739 to MF, and 491 to CC. Within the 995 up-regulated DEGs, 295 corresponded to BP, 352 to MF, and 239 to CC, while among the 1342 down-regulated DEGs, 341 belonged to BP, 387 to MF, and 252 to CC (Figure S1A). Overall, the number of functionally annotated DEGs across all three GO categories was markedly higher in the ART vs. CK than in the PHY vs. CK comparison, indicating a more extensive metabolic perturbation induced by zooplankton feeding.

**Figure 5:**
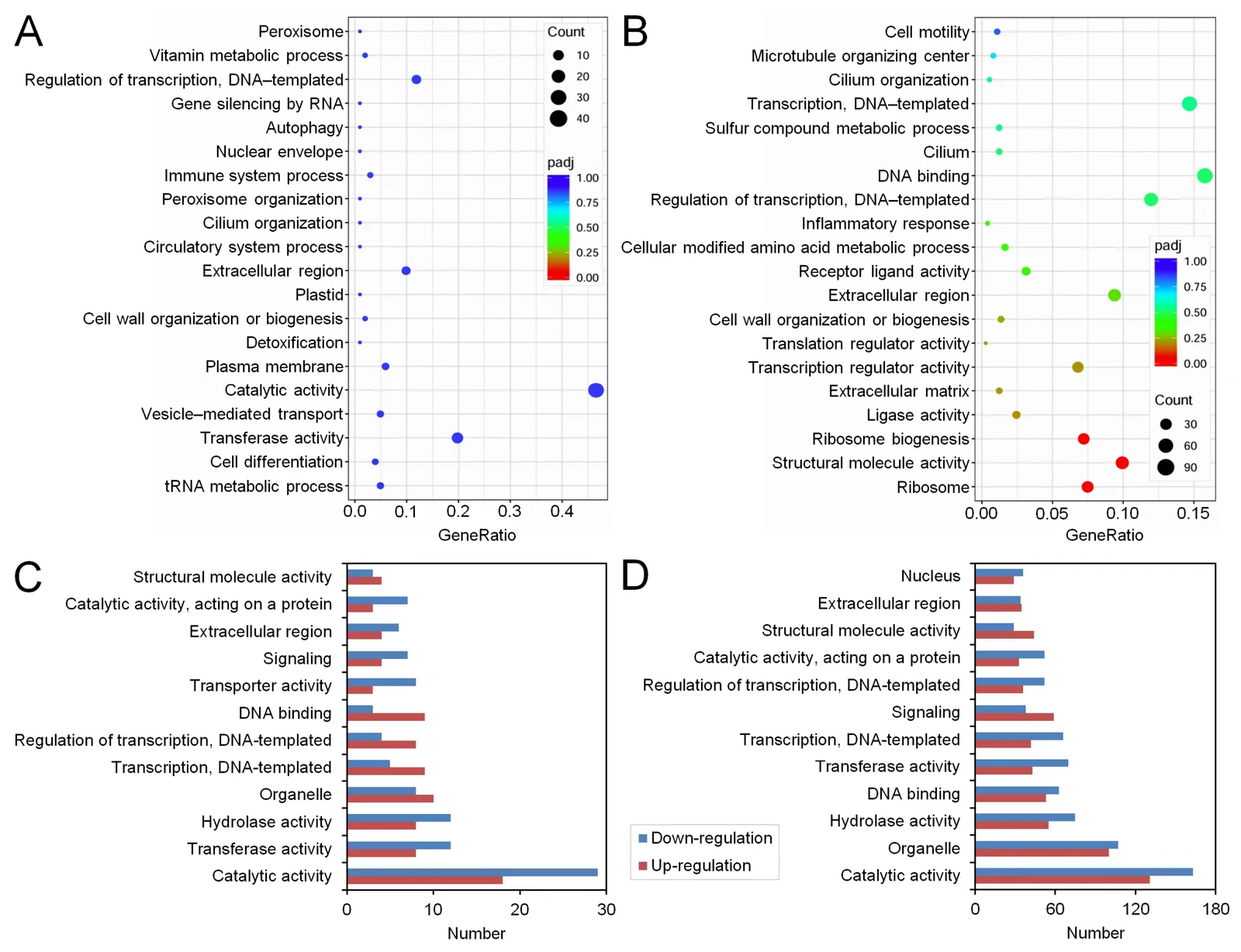
Gene Ontology (GO) functional enrichment analysis of *Craspedacusta sowerbii* under different prey conditions. (**A**,**B**) Overview of enriched GO terms in phytoplankton-feeding groups compared with control groups with no prey supplementation (PHY vs. CK) and zooplankton-feeding groups compared with control groups with no prey supplementation (ART vs. CK), respectively. (**C**,**D**) Top 12 GO terms ranked by total differentially expressed gene numbers in the PHY vs. CK and ART vs. CK comparisons, respectively. padj, adjusted *p*.

No significantly enriched GO terms were detected in the PHY vs. CK comparison, neither in the overall enrichment view nor in separate analyses of up- or down-regulated gene sets (Figure 5A, File S3). In contrast, the ART vs. CK comparison exhibited three significantly up-regulated enriched GO terms, including ribosome (55 DEGs, padj = 0.0027), structural molecule activity (73 DEGs, padj = 0.0028), and ribosome biogenesis (53 DEGs, padj = 0.0030) (Figure 5B). Among them, 40 DEGs were shared between ribosome biogenesis and ribosome (padj = 3 × 10^−7^ for both terms), and 44 DEGs were annotated to structural molecule activity (padj = 1.9 × 10^−4^) (File S3).

GO terms from each comparison were ranked by enrichment significance, and the top 12 up- and down-regulated pathways were summarized (Figure S1B–E). Given the absence of significant GO enrichment in the PHY vs. CK comparison, functional pathways were further ranked according to DEG abundance to compare functional perturbation between treatments. In the PHY vs. CK comparison, the top three GO terms with the most DEG counts were catalytic activity (47 DEGs, 18 up-regulated, 29 down-regulated), transferase activity (20 DEGs, 8 up-regulated, 12 down-regulated), and hydrolase activity (20 DEGs, 8 up-regulated, 12 down-regulated) (Figure 5C). In the ART vs. CK comparison, the most abundant functional terms were catalytic activity (294 DEGs, 131 up-regulated, 163 down-regulated), organelle (207 DEGs, 100 up-regulated, 107 down-regulated), and hydrolase activity (130 DEGs, 55 up-regulated, 75 down-regulated) (Figure 5D). The two shared functional categories (catalytic activity and hydrolase activity) exhibited substantially greater DEG abundance and broader regulatory variation in the ART vs. CK than in the PHY vs. CK comparisons, demonstrating that zooplankton feeding triggers more complex and intensive metabolic regulation in *C. sowerbii*.

**Figure 6:**
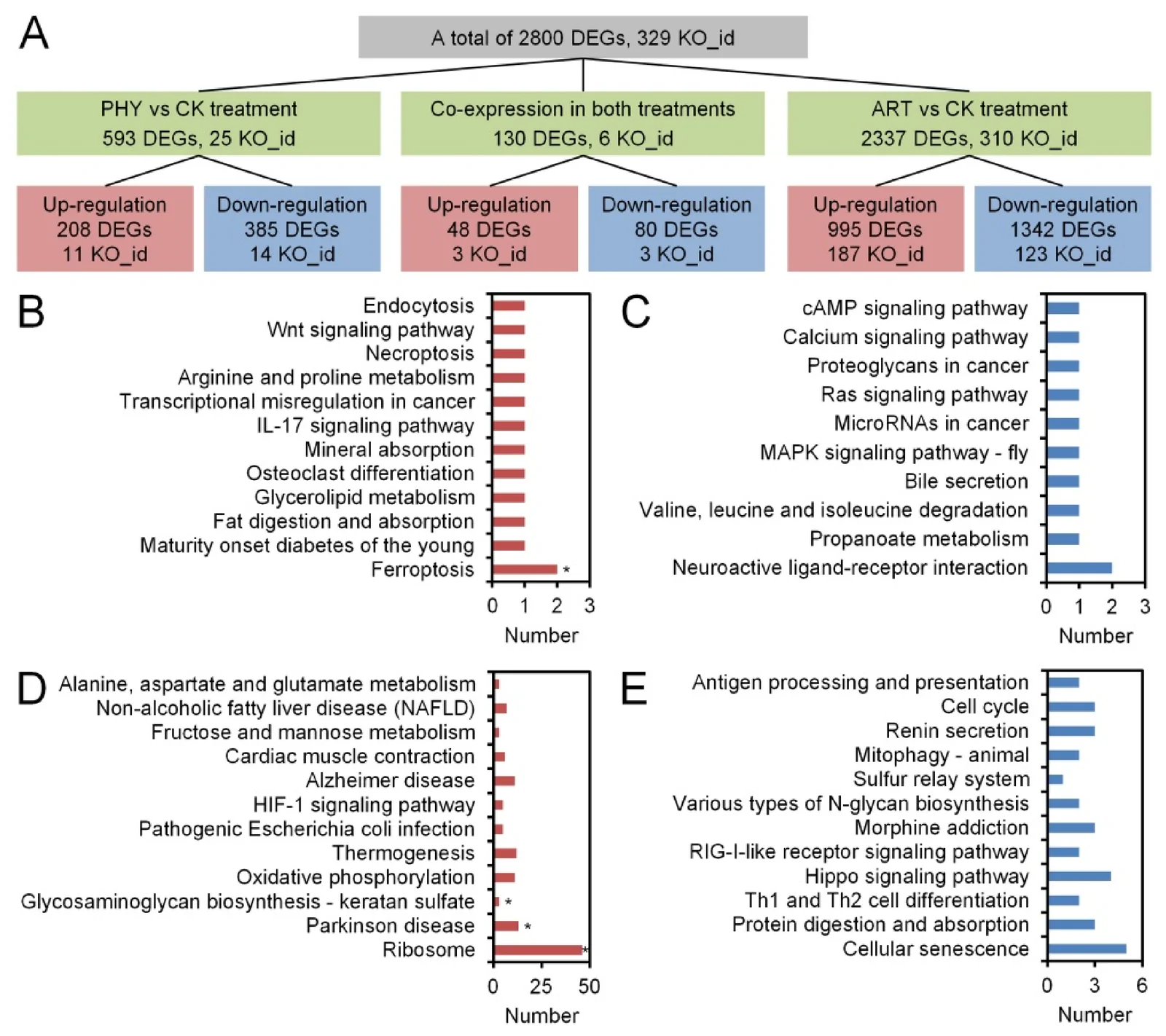
Kyoto Encyclopedia of Genes and Genomes (KEGG) pathway enrichment of differentially expressed genes (DEGs) from phytoplankton and zooplankton feeding treatments. (**A**) Statistical overview of KO-annotated DEGs in different comparisons. (**B**,**C**) Top 12 up- and down-regulated KEGG pathways in phytoplankton-feeding groups compared with control groups with no prey supplementation (PHY vs. CK). (**D**,**E**) Top 12 up- and down-regulated KEGG pathways in zooplankton-feeding groups compared with control groups with no prey supplementation (ART vs. CK). Pathways with significant enrichment (padj ≤ 0.05) are marked with asterisks (*).

Consistent with GO annotation, KEGG pathway analysis further revealed distinct metabolic responses between the two feeding treatments. Among the 2800 total DEGs, 329 were successfully annotated with KEGG Orthology identifiers (KO_id). In the PHY vs. CK comparison, only 25 DEGs were assigned KO_id, including 11 up-regulated and 14 down-regulated genes (Figure 6A, File S2). In comparison, 310 DEGs in the ART vs. CK comparison received KO_id, consisting of 187 up-regulated and 123 down-regulated genes (Figure 6A, File S2), confirming that zooplankton feeding induces far more extensive metabolic modulation than phytoplankton feeding.

KEGG pathways of each comparison were ranked by enrichment significance, and the top 12 up- and down-regulated pathways were screened for functional characterization. In the PHY vs. CK comparison, only the ferroptosis pathway was significantly up-regulated (ko04216, 2 DEGs, padj = 0.0044); no pathways were significantly down-regulated (Figure 6B,C, File S4). In the ART vs. CK comparison, three pathways exhibited significant up-regulation, including ribosome (ko03010, 46 DEGs, padj = 2.49 × 10^−19^), Parkinson disease (ko05012, 13 DEGs, padj = 0.0398), and glycosaminoglycan biosynthesis–keratan sulfate (ko00533, 3 DEGs, padj = 0.0458) (Figure 6D, File S4). Two additional metabolic pathways did not reach the threshold for significant differential expression yet showed strong biological relevance, namely oxidative phosphorylation (ko00190, 11 DEGs, padj = 0.0794) and thermogenesis (ko04714, 12 DEGs, padj = 0.1857) (Figure 6D, File S4). No significantly down-regulated KEGG pathways were identified in the ART vs. CK comparison (Figure 6E, File S4). Full pathway rankings for both comparisons are provided in Figure S2.

### 3.5. Alterations in Gene Expression Profiles of C. sowerbii Fed with Phytoplankton or Zooplankton

Integrating the behavioral observations and transcriptomic results, we speculated that the metabolic alterations induced by zooplankton feeding constitute the core molecular mechanism underlying the feeding preference of *C. sowerbii*. Since these key metabolic pathways were predominantly enriched in the up-regulated gene sets of the ART vs. CK comparison, we further characterized the functional features and expression patterns of these differentially regulated KEGG pathways, including ribosome, Parkinson’s disease, glycosaminoglycan biosynthesis–keratan sulfate, oxidative phosphorylation, and thermogenesis, along with other biologically relevant pathways.

Transcriptomic profiling identified a total of 46 genes associated with ribosome function. None of these genes were differentially expressed in the PHY vs. CK comparison, whereas all were significantly up-regulated in the ART vs. CK comparison (Figure 6D, Files S2 and S4). Six ribosome-related genes with the lowest *p*-values were selected as representative candidates, including Cluster-2809.0, Cluster-2727.0, Cluster-2890.0, Cluster-2811.0, Cluster-2396.0, and Cluster-2731.0. All of them exhibited log2FC values greater than 6 (Table 1, File S2), indicating that the protein synthesis machinery of *C. sowerbii* was strongly activated upon zooplankton ingestion.

A set of genes involved in the mitochondrial electron transport chain (ETC) and energy metabolism were significantly up-regulated in the ART vs. CK comparison. Specifically, five genes (Cluster-10902.59080, Cluster-10902.7998, Cluster-10902.7759, Cluster-2889.0, and Cluster-1803.0) encode key structural subunits of mitochondrial ETC Complex I, including NADH ubiquinone oxidoreductase chains 2–5 (ND2–5) and NADH dehydrogenase 1 alpha subcomplex subunit 4 (NDUFA4), all of which showed high log2FC values (Table 1, File S2). Cluster-10902.61929 encodes cytochrome b (CYTB), a core component of ETC Complex III, with a log2FC value of 13.85 (Table 1, File S2). Three additional genes (Cluster-10902.59421, Cluster-2781.0, and Cluster-1171.0) encode cytochrome c oxidase subunits 3, 4, and 8 (COX3, COX4, and COX8) of ETC Complex IV, with consistently up-regulated expression. Moreover, Cluster-2753.0 and Cluster-2817.0 encode F-type H^+^-transporting ATPase subunits c and beta (ATPeF0C, ATPeF1B), structural components of ETC Complex V, which yielded log2FC values above 5 (Table 1, File S2). Collectively, these 11 ETC-related genes were centrally involved in multiple up-regulated metabolic pathways in the ART vs. CK comparison, including oxidative phosphorylation, thermogenesis, and Parkinson’s disease, as well as cardiac muscle contraction and non-alcoholic fatty liver disease (Figure 6D), implying that activation of mitochondrial ETC assembly and ATP synthesis constitutes a critical physiological response of *C. sowerbii* upon zooplankton feeding.

Several functional genes related to prey adhesion and tissue remodeling also exhibited significant up-regulation in the ART vs. CK comparison. Three genes (Cluster-19509.0, Cluster-10902.5739, and Cluster-16739.0) involved in glycosaminoglycan biosynthesis–keratan sulfate showed mild up-regulation (Table 1, File S2). Five genes (Cluster-10005.1, Cluster-2652.0, Cluster-2652.1, Cluster-1601.0, and Cluster-2752.0) encode tubulin beta (TUBB) and Ras homolog family member A (RHOA), two master regulators of cytoskeletal assembly and cellular motility, which were markedly up-regulated (Table 1, File S2). Another five genes (Cluster-1567.0, Cluster-2680.0, Cluster-18403.0, Cluster-15678.0, and Cluster-1949.0) were associated with a stress response that was enriched in the HIF-1 signaling pathway and showed consistent up-regulation (Table 1, File S2). Three genes (Cluster-1567.0, Cluster-2591.0, and Cluster-2829.0) participated in fructose and mannose metabolism. Another three genes (Cluster-3027.0, Cluster-2013.0, and Cluster-10902.21421) were involved in alanine, aspartate, and glutamate metabolism. All of these genes were significantly up-regulated in the ART vs. CK comparison (Table 1, File S2), confirming that zooplankton feeding induces comprehensive metabolic reprogramming in *C. sowerbii*.

## 4. Discussion

### 4.1. The Molecular Basis of Feeding Preference in Craspedacusta sowerbii

Predator–prey interactions represent the core driver of material cycling and energy transfer in aquatic food webs; such trophic relationships further regulate community diversity and ecosystem stability [41]. For aquatic IAS, feeding strategy and nutrient assimilation are critical determinants of their habitat adaptability and invasive success [42,43]. Selective feeding is a widespread adaptive trait among aquatic organisms, driven by complex physiological, biochemical, and molecular regulatory processes [44,45]. Previous investigations have demonstrated that most cnidarians exhibit pronounced selective predation [46,47]. The freshwater jellyfish *Limnocnida tanganjicae* displays a distinct feeding preference for copepods [48], and the planktonic cnidarian *Odessia maeotica* can substantially reshape zooplankton community structure and alter aquatic trophic dynamics [49]. In line with these findings, studies have confirmed that *C*. *sowerbii* exhibits prominent prey selectivity, with a strong feeding bias toward zooplankton over phytoplankton [35,50]. Despite this well-documented behavioral evidence, the physiological regulatory strategies and molecular basis underlying prey selection in *C. sowerbii* remain largely uncharacterized. Most previous investigations relied solely on field ecological surveys or isolated behavioral descriptions, whereas experimental evidence at the molecular level for interpreting prey discrimination has been lacking. To our knowledge, this study represents the first integration of controlled behavioral assays and comparative transcriptomics to dissect the mechanistic basis of prey selectivity in adult *C. sowerbii* medusae. Our work thus moves beyond descriptive feeding observations and provides direct molecular evidence explaining why this invasive jellyfish discriminates between zooplankton and phytoplankton prey.

By feeding *C. sowerbii* with zooplankton and phytoplankton separately in an indoor culture system (Figure 1), the present study integrated behavioral observations and comparative transcriptomics to reveal the multi-layered mechanisms of prey preference in *C. sowerbii*. Behavioral observations demonstrated clear divergent feeding responses of *C. sowerbii* to different prey (Figure 2 and Figure 3); transcriptomic profiling revealed distinct metabolic responses between the two treatments (Figure 3, Figure 4, Figure 5 and Figure 6). These results demonstrate that *C. sowerbii* can precisely discriminate prey types and initiate specific physiological programs. Gene Ontology (GO) functional analysis indicated that the differentially expressed genes (DEGs) associated with catalytic activity and hydrolase activity were substantially enriched in zooplankton-feeding groups compared with control groups with no prey supplementation (ART vs. CK) and in phytoplankton-feeding groups compared with control groups with no prey supplementation (PHY vs. CK) (Figure 5), indicating *C. sowerbii* possesses a sophisticated molecular regulatory network specifically activated for zooplankton digestion.

Phytoplankton and zooplankton differ markedly in their biochemical composition, a distinction that is primarily manifested in their structural barriers to hydrolytic digestion, as evidenced by the differential responses of *C. sowerbii* to algae and *Artemia* nauplii (Figure 3). Algal cell walls are primarily composed of structural polysaccharides such as cellulose and pectin, which serve as a primary defensive barrier that restricts predators’ access to intracellular nutrients [51]. In contrast, the exoskeleton of *Artemia* nauplii consists mainly of a chitin–protein complex, which constitutes the dominant physical obstacle during digestive hydrolysis [52]. Consequently, predators must produce corresponding hydrolases to disrupt these barriers (cellulose for algae and chitin for zooplankton) to achieve efficient nutrient assimilation. Transcriptomic profiling of *C. sowerbii* revealed abundant transcripts encoding proteases, chitinases, trypsins, peptidases, and lipases, whereas no homologous genes for cellulase or pectinase were detected (File S1). It should be noted that genes encoding these hydrolases were only identified in the transcript pool and not among DEGs, owing to the fact that the digestion process was largely completed at the sampling time point. This repertoire of proteases, chitinases, and trypsins enables *C. sowerbii* to efficiently degrade the chitinous exoskeleton of ingested *Artemia* [53], consistent with the morphological observations of extensive degradation within the gastric cavity (Figure 3). Conversely, the complete absence of cellulase and pectinase transcripts renders *C. sowerbii* incapable of dismantling the polysaccharide-rich cell walls of algae [54], thereby limiting its digestive efficiency on phytoplankton (Figure 3). Collectively, the transcriptional divergence in hydrolase genes underlies differential catabolic capacities toward phytoplankton and zooplankton, constituting a key molecular mechanism driving the selective feeding behavior of *C. sowerbii*.

KEGG enrichment further identified ribosome biogenesis, oxidative phosphorylation, and thermogenesis as the most significantly activated pathways in response to zooplankton predation (Figure 6). These pathways dominate core cellular biosynthesis and energy metabolism, which precisely match the high energy demands of active predation, locomotion, and digestive metabolism [55,56]. The significant up-regulation of ETC Complex I–V subunit genes verified enhanced mitochondrial respiration and ATP production under zooplankton-feeding conditions (Table 1), providing essential energy support for the entire predation process of *C. sowerbii*, including prey searching, capture, encapsulation, and continuous nutrient assimilation (Figure 2 and Figure 3). Notably, the absence of such metabolic activation under phytoplankton treatment explains the failure of phytoplankton utilization and the passive feeding phenotype observed in this study (Figure 5 and Figure 6, Files S2–S4).

Beyond core energy metabolism, multiple functional pathways associated with predation adaptation were specifically activated during zooplankton feeding. The up-regulation of glycosaminoglycan biosynthesis pathways indicated enhanced mucus synthesis and secretion, which is a crucial auxiliary strategy for prey adhesion and capture in cnidarians [57,58]. Meanwhile, the elevated expression of TUBB and RHOA, which are involved in cytoskeletal regulation, reflected enhanced cell motility and body deformation capacity, which underpins the active predatory locomotion of *C. sowerbii* during zooplankton capture [59]. In addition, the activation of HIF-1 signaling and carbohydrate metabolism pathways reflected adaptive metabolic remodeling and mild postprandial stress regulation, enabling *C. sowerbii* to maintain physiological homeostasis during high-intensity predation and digestion [60]. Collectively, the coordinated activation of cytoskeletal movement, mucus secretion, energy supply, and homeostatic regulation forms a complete physiological regulatory cascade that supports the specialized zooplankton-preference feeding strategy of *C. sowerbii*. This physiological remodeling observed in *C. sowerbii* is highly consistent with the thermic effect of food (TEF). TEF represents the mandatory extra energy consumption during postprandial digestion, nutrient absorption, and anabolism, which is widely documented in vertebrates with distinct interspecific regulatory characteristics [61,62]. Here, we identified a typical TEF-like transcriptional metabolic response induced by zooplankton ingestion in *C. sowerbii*, accounting for its robust activation of energy metabolism toward zooplankton but minimal metabolic perturbation when encountering phytoplankton.

### 4.2. Potential Role of Algicidal Bacteria in the Gastric Cavity of C. sowerbii

Robust transcriptional activation of hydrolytic enzymes, ribosome biogenesis and energy metabolism constitutes the key molecular basis for efficient nutrient utilization of zooplankton by *C. sowerbii*. For *C. sowerbii*, an evolutionarily basal invertebrate, a key unresolved issue concerns whether it can acquire and exploit phytoplankton-derived nutrition under conditions of low or total absence of zooplankton prey. To clarify the in situ feeding characteristics of *C. sowerbii* under natural conditions, we previously investigated the dietary composition of reservoir-cultured individuals through gastric content dissection and amplicon sequencing [16]. Building on the findings of the present study, we further conducted a more detailed reanalysis of the field observations.

**Figure 7:**
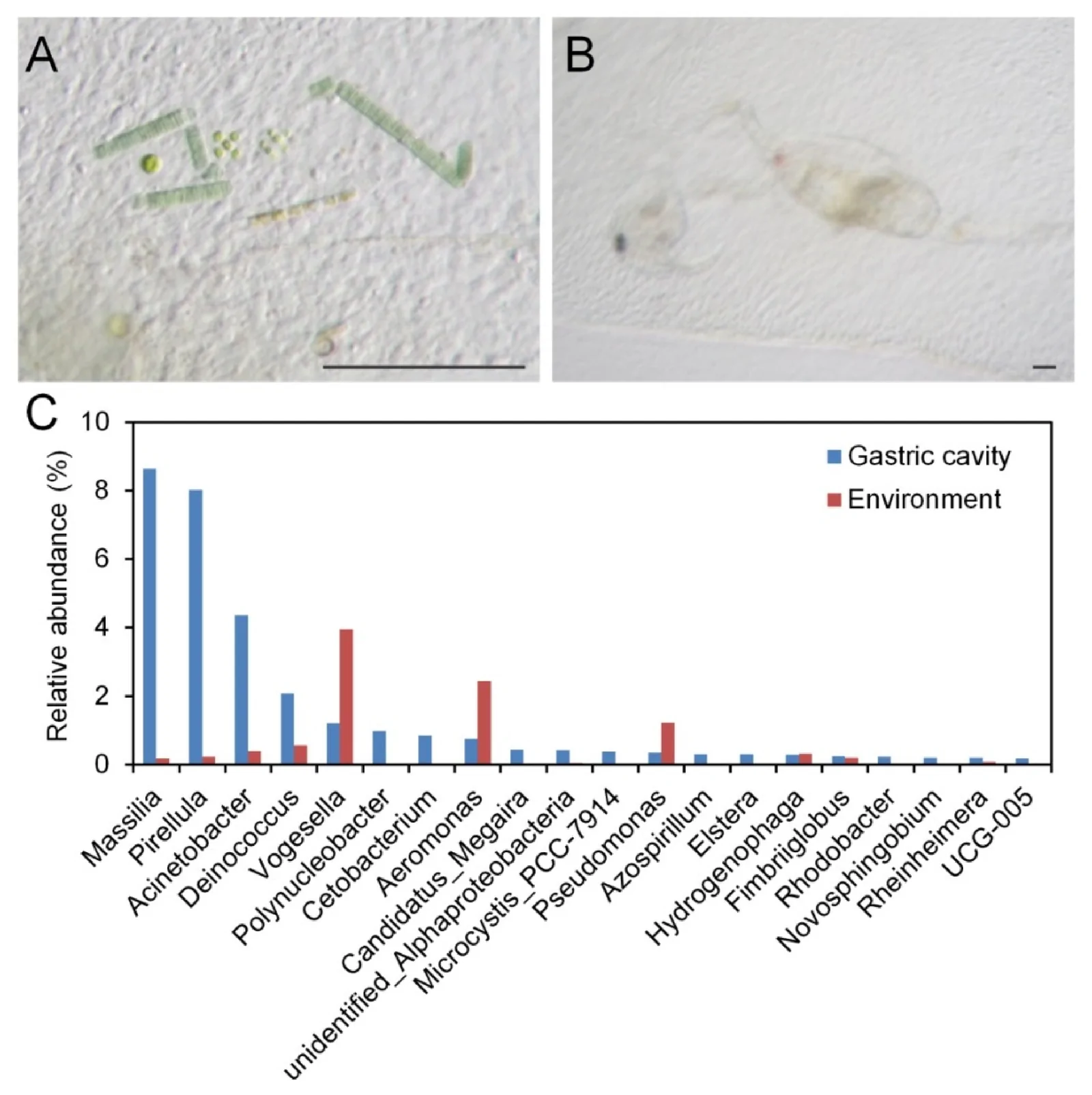
Dietary composition of reservoir-cultured *Craspedacusta sowerbii*. (**A**,**B**) Observations showing phytoplankton and zooplankton present in the gastric cavity of reservoir-cultured *C. sowerbii*. (**C**) Genus-level composition of functional microorganisms in the gastric cavity of *C. sowerbii*; data were derived from re-analysis of 16S rRNA amplicon sequencing data originally published in reference [16]. Bars = 100 μm.

Our results revealed that the gastric cavity of reservoir-cultured *C. sowerbii* harbors both phytoplankton and zooplankton. Specifically, most ingested phytoplankton cannot be digested (Figure 7A), whereas consumed zooplankton are readily digestible (Figure 7B). Importantly, building upon 16S rRNA amplicon sequencing data published in our previous study [16], we performed a dedicated re-analysis comparing microbial communities in the gastric cavity of *C. sowerbii* after 5 days’ in situ acclimation against the microbial assemblages in surrounding water (Figure 7C, File S5). This comparative analysis reveals significant enrichment of distinct microbial taxa within the gastric cavity, a component that has been largely neglected in previous studies. Among the five most abundant genera identified, *Massilia*, *Pirellula*, *Acinetobacter*, and *Deinococcus* exhibited distinctly higher relative abundances in the gastric cavity than in the surrounding environment. Notably, several of these enriched taxa, including *Massilia*, *Acinetobacter* and *Deinococcus*, have been well documented as typical algicidal bacteria in aquatic ecosystems [63].

Symbiotic microbial-assisted digestion of refractory organic substances is a pervasive adaptive strategy across diverse higher organisms [64,65]. Host-associated gut microbiomes confer multiple ecological advantages, including facilitating environmental acclimation, decomposing indigestible dietary components, degrading harmful toxins, and resisting parasitic invasion [66]. Ecologically, algicidal bacteria represent a key functional microbial group capable of suppressing algal proliferation, lysing algal cells, and degrading algal biomass, thereby modulating phytoplankton abundance and community structure in aquatic systems [63,67].

Given the substantial accumulation of undigested phytoplankton biomass within the gastric cavity of *C. sowerbii*, a key question arises as to whether these entrapped phytoplankton are merely excreted without nutritional assimilation. If *C. sowerbii* inherently lacks the capacity to digest and utilize phytoplankton, the passive ingestion and accumulation of algal cells would appear evolutionarily counterintuitive. This apparent paradox strongly suggests that *C. sowerbii* may possess uncharacterized, latent nutritional strategies for exploiting phytoplankton resources. We therefore hypothesize that gastric-enriched algicidal bacteria and other symbiotic microbes assist *C. sowerbii* in breaking down phytoplankton biomass under natural conditions, thereby enabling indirect utilization of phytoplankton-derived nutrients. This host–microbe synergistic nutritional hypothesis offers a new conceptual framework to partially explain the broad environmental adaptability and invasive success of *C. sowerbii*, and such a microbe-mediated trophic strategy may serve as an alternative route for nutrient acquisition, enhancing resource-use efficiency and ecological resilience under prey-deficient conditions. Notably, this strategy was not observed in our indoor artificial culture system, which is most likely attributable to the absence of the diverse and abundant environmental microorganisms present in natural aquatic habitats. Follow-up systematic research is therefore warranted to validate this synergistic host–microbe nutritional interaction.

From an invasion-management perspective, our mechanistic findings deliver practical implications for risk assessment and mitigation of *C. sowerbii* outbreaks. Since medusae strongly depend on zooplankton for nutrition, reservoir and lake management strategies targeting the zooplankton community may help constrain population proliferation of this invasive species. Nevertheless, we should also keep in mind the potential auxiliary nutrition supplied by gastric symbiotic algicidal bacteria under zooplankton-scarce conditions, which may sustain jellyfish survival even when zooplankton prey become limited. Therefore, plankton-based mitigation measures should consider both prey availability and host-microbe interactions, rather than only focusing on zooplankton abundance.

Although our integrated behavioral-transcriptomic approach has revealed key mechanistic insights into prey preference of *C. sowerbii*, several experimental limitations remain to be addressed in future work and should be acknowledged. Importantly, these constraints do not invalidate our major conclusions. First, *C. sowerbii* is an understudied non-model hydrozoan lacking established genetic manipulation and long-term rearing protocols [68]. The delicate morphology of medusae results in very low tissue biomass, which imposes constraints on parallel multi-omics validation and functional genetic characterization. In the present experimental design, all collected specimens were allocated to RNA library preparation to capture global transcriptional profiles, and thus RT-PCR validation of the RNA-seq results was not conducted. Independent transcriptional assays are still required to corroborate the perturbed metabolic pathways identified herein. Second, the prey assemblages deployed in our experiments lack broad ecological representativeness. Natural freshwater systems host a wide spectrum of diatoms, cyanobacteria, rotifers, cladocerans and copepods; additional trials incorporating ecologically dominant prey taxa are needed to test the universal applicability of our observed feeding responses. Third, fixed temperature and photoperiod regimes disregarded natural diel and seasonal oscillations that modulate the feeding and metabolic rhythms of naturally occurring medusae. Lastly, this study exclusively focused on the free-swimming medusa stage, whereas the benthic polyp phase was not investigated. Potential disparities in trophic regulation across life-cycle stages therefore remain to be characterized. Importantly, these limitations do not undermine our core discoveries, which for the first time unravel the multi-tiered machinery underlying the specialized zooplankton predation strategy of *C. sowerbii*.

## 5. Conclusions

In this study, we combined controlled behavioral observations and comparative transcriptomics to systematically investigate prey-preference mechanisms in the globally invasive freshwater jellyfish *Craspedacusta sowerbii*. Our behavioral experiments confirm that medusae actively hunt and efficiently digest zooplankton within 6 h, whereas phytoplankton are only passively ingested and remain undigested. Zooplankton ingestion triggers extensive transcriptional reprogramming, including activation of digestive hydrolysis, ribosome biogenesis and mitochondrial energy-metabolism pathways. In contrast, phytoplankton exposure induces negligible transcriptomic alteration; the divergent digestive capacity is partly explained by the differential deployment of hydrolase-related gene repertoires.

This work makes two key contributions to freshwater invasion ecology and cnidarian trophic physiology. First, to our knowledge, this is the first mechanistic study integrating feeding behavior and transcriptome to decipher prey discrimination in *C. sowerbii*, filling a long-standing knowledge gap regarding the molecular basis of its trophic selectivity. Second, our study raises a testable hypothesis that gastric symbiotic algicidal bacteria may enable indirect phytoplankton utilization under natural environments, highlighting the underappreciated role of host-microbe interactions in the survival strategy of this invasive jellyfish. Collectively, these findings advance current understanding of how physiological and molecular processes shape feeding selectivity and invasive fitness of *C. sowerbii*. The results also provide mechanistic support for risk evaluation and management considerations for this globally spreading hydrozoan. Further multi-omics, microbial manipulation and life-cycle-wide experiments are required to validate the microbe-assisted nutritional hypothesis and generalize these findings across variable natural environmental conditions.

## Figures and Tables

**Table 1:** Significantly up-regulated differentially expressed genes (DEGs) in zooplankton-feeding groups compared with control groups with no prey supplementation (ART vs. CK). Gene_id, a unique gene identifier generated after de-redundancy using Corset. KO_id, Kyoto Encyclopedia of Genes and Genomes Orthology identifier. KO name, Kyoto Encyclopedia of Genes and Genomes Orthology name. log2FC, log2 Fold Change. pval, *p*-value.

Gene_id	KO_id	KO Name	log2FC	pval
Cluster-2809.0	K02943	RP-LP2, RPLP2	7.46	2.15 × 10^−7^
Cluster-2727.0	K02951	RP-S12e, RPS12	6.74	1.97 × 10^−5^
Cluster-2890.0	K02880	RP-L17e, RPL17	6.72	3.74 × 10^−5^
Cluster-2811.0	K02955	RP-S14e, RPS14	6.57	3.91 × 10^−5^
Cluster-2396.0	K02969	RP-S20e, RPS20	6.49	5.03 × 10^−5^
Cluster-2731.0	K02993	RP-S7e, RPS7	6.53	7.08 × 10^−5^
Cluster-10902.59080	K03879	ND2	19.91	6.31 × 10^−33^
Cluster-10902.7998	K03880	ND3	10.29	2.10 × 10^−7^
Cluster-10902.7759	K03881	ND4	13.27	7.29 × 10^−10^
Cluster-2889.0	K03883	ND5	13.47	1.30 × 10^−9^
Cluster-1803.0	K03948	NDUFA4	4.58	4.07 × 10^−2^
Cluster-10902.61929	K00412	CYTB, petB	13.85	3.53 × 10^−11^
Cluster-10902.59421	K02262	COX3	14.76	1.52 × 10^−13^
Cluster-2781.0	K02263	COX4	4.66	1.40 × 10^−2^
Cluster-1171.0	K02273	COX8	4.57	3.60 × 10^−2^
Cluster-2753.0	K02128	ATPeF0C, ATP5G, ATP9	7.40	3.00 × 10^−3^
Cluster-2817.0	K02133	ATPeF1B, ATP5B, ATP2	5.87	5.27 × 10^−4^
Cluster-19509.0	K01022	CHST1	1.40	1.26 × 10^−2^
Cluster-10902.5739	K09671	CHST6	1.07	3.91 × 10^−3^
Cluster-16739.0	K07966	B4GALT1	1.02	3.98 × 10^−3^
Cluster-10005.1	K07375	TUBB	1.20	1.76 × 10^−2^
Cluster-2652.0	K07375	TUBB	7.37	1.33 × 10^−3^
Cluster-2652.1	K07375	TUBB	4.44	4.24 × 10^−2^
Cluster-1601.0	K07375	TUBB	4.47	4.97 × 10^−2^
Cluster-2752.0	K04513	RHOA	5.64	1.75 × 10^−3^
Cluster-1567.0	K01623	ALDO	4.88	2.34 × 10^−2^
Cluster-2680.0	K00134	GAPDH, gapA	5.42	3.03 × 10^−3^
Cluster-18403.0	K09592	EGLN, HPH	2.21	4.96 × 10^−3^
Cluster-15678.0	K09592	EGLN, HPH	2.10	2.63 × 10^−6^
Cluster-1949.0	K02991	RP-S6e, RPS6	6.33	1.17 × 10^−4^
Cluster-1567.0	K01623	ALDO	4.88	2.34 × 10^−2^
Cluster-2591.0	K00966	GMPP	7.22	6.43 × 10^−3^
Cluster-2829.0	K00966	GMPP	4.97	4.84 × 10^−3^
Cluster-3027.0	K13566	NIT2, yafV	4.87	2.00 × 10^−2^
Cluster-2013.0	K01915	glnA, GLUL	4.48	2.48 × 10^−2^
Cluster-10902.21421	K14592	FOLH1, GCPII	1.06	9.62 × 10^−4^

## Data Availability

The data are contained within the article.

## Supplementary Materials

The following supporting information can be downloaded at: https://www.mdpi.com/article/10.3390/biology15212002/s1, Figure S1. Gene Ontology (GO) functional enrichment analysis of *Craspedacusta sowerbii* under different prey conditions. (**A**) Statistical overview of GO functional annotation across different comparisons. (**B**,**C**) Top 12 most significant up- and down-regulated Go terms in phytoplankton‑feeding groups compared with control groups with no prey supplementation (PHY vs. CK). (**D**,**E**) Top 12 most significant up- and down-regulated Go terms in zooplankton‑feeding groups compared with control groups with no prey supplementation (ART vs. CK). Go terms with significant enrichment (adjusted *p* ≤ 0.05) are marked with asterisks (*). Figure S2. Kyoto Encyclopedia of Genes and Genomes (KEGG) pathway enrichment of *Craspedacusta sowerbii* under different prey conditions. (**A**,**B**) Overview of KEGG enrichment in phytoplankton‑feeding groups compared with control groups with no prey supplementation (PHY vs. CK) and zooplankton‑feeding groups compared with control groups with no prey supplementation (ART vs. CK), respectively. File S1, Co-expression genes of the three treatments. File S2, Differentially expressed genes of different comparisons. File S3, Gene Ontology enrichment of differentially expressed genes. File S4, Kyoto Encyclopedia of Genes and Genomes enrichment of differentially expressed genes. File S5, 16S rRNA amplicon sequencing derived from [16].
