# Supplementary material for: Prey Preference of the Freshwater Jellyfish Craspedacusta sowerbii: Integrated Behavioral Observations and Transcriptomic Responses to Phytoplankton and Zooplankton
Source: Biology (Basel). 2026 Nov 4;15(21):2002. doi: 10.3390/biology15212002 (PMC13761970; doi:10.3390/biology15212002)
Supplement: Supplementary file 1 [file biology-15-02002-s001.zip › Supplementary figures.pdf]

Article

Prey Preference of the Freshwater Jellyfish *Craspedacusta sowerbii*: Integrated Behavioral Observations and Transcriptomic Responses to Phytoplankton and Zooplankton

Hailong Yan [1,*], Yu Wang [2], Lin Li [1], Yufan He [1], Jinxiang Han [1], Kefei Zhu [1], Yingchun Gong [3,*], Nicola Fohrer [1,4], Jianguang Qin [5] and Yuying Li [1]

[1] International Joint Laboratory of Watershed Ecological Security for Water Source Region of Middle Route Project of South-North Water Diversion in Henan Province, College of Water Resource and Modern Agriculture, Nanyang Normal University, Nanyang 473061, China; lilin130719@163.com (L.L.); 15637509979@163.com (Y.H.); hanjinxiang2026@163.com (J.H.); 13673396417@163.com (K.Z.); nfohrer@hydrology.uni-kiel.de (N.F.); lyying200508@163.com (Y.L.)

[2] Henan Academy of Fishery Sciences, Henan Academy of Agricultural Sciences, Zhengzhou 450044, China; wangyu@hnagri.org.cn

[3] Institute of Hydrobiology, Chinese Academy of Sciences, Wuhan 430072, China

[4] Department of Hydrology and Water Resources Management, Kiel University, 240980 Kiel, Germany

[5] College of Science and Engineering, Flinders University, Adelaide, SA 5001, Australia; jian.qin@flinders.edu.au

* Correspondence: hailong@nynu.edu.cn (H.Y.); springgong@ihb.ac.cn (Y.G.)

Supplementary Figures

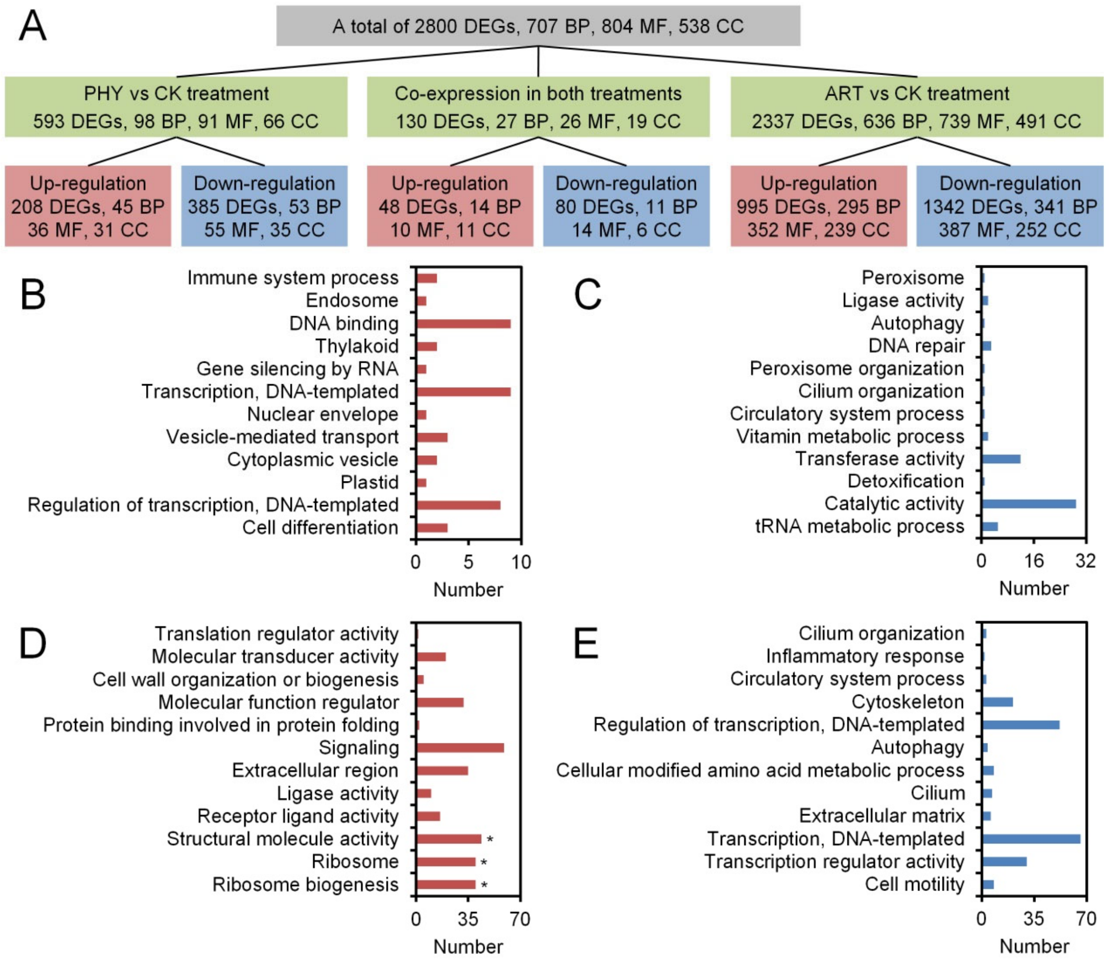

Figure S1. Gene Ontology (GO) functional enrichment analysis of *Craspedacusta sowerbii* under different prey conditions. (**A**) Statistical overview of GO functional annotation across different comparisons. (**B**,**C**) Top 12 most significant up- and down-regulated Go terms in phytoplankton-feeding groups compared with control groups with no prey supplementation (PHY vs. CK). (**D**,**E**) Top 12 most significant up- and down-regulated Go terms in zooplankton-feeding groups compared with control groups with no prey supplementation (ART vs. CK). Go terms with significant enrichment (adjusted $p \leq 0.05$) are marked with asterisks (*).

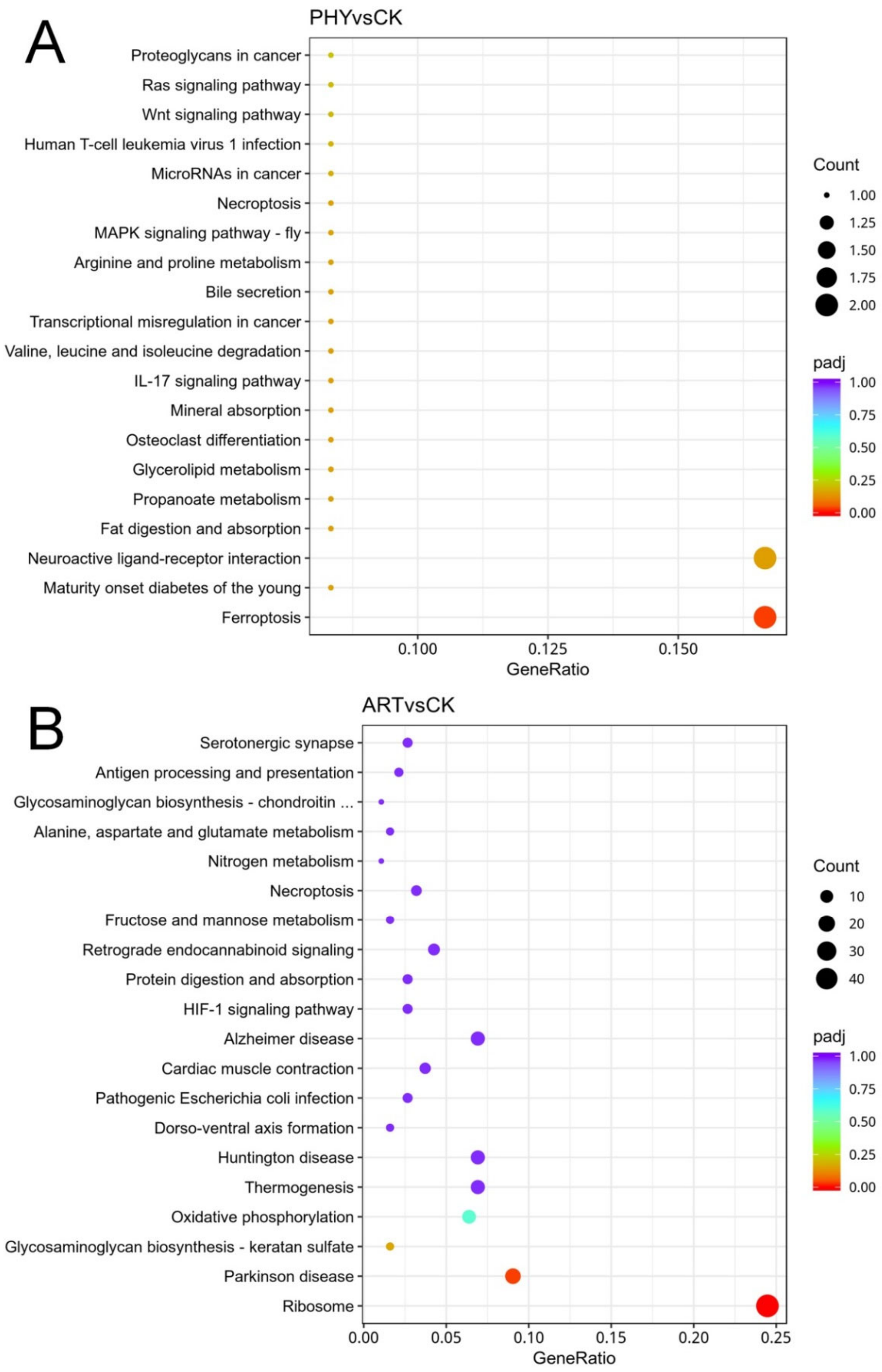

Figure S2. Kyoto Encyclopedia of Genes and Genomes (KEGG) pathway enrichment of *Craspedacusta sowerbii* under different prey conditions. (**A**,**B**) Overview of KEGG enrichment in phytoplankton-feeding groups compared with control groups with no prey supplementation (PHY vs. CK) and zooplankton-feeding groups compared with control groups with no prey supplementation (ART vs. CK), respectively.
